# Antimicrobial resistance in Salmonella enteritidis, southern Italy, 1990-1998.

**DOI:** 10.3201/eid0604.000415

**Published:** 2000

**Authors:** A Nastasi, C Mammina, L Cannova

**Affiliations:** University of Florence, Florence, Italy.

## Abstract

During 1990 to 1998, we identified multidrug-resistant isolates of Salmonella Enteritidis in southern Italy. Plasmids containing class I integrons and codifying for synthesis of extended- spectrum beta-lactamases were detected. Active surveillance for resistance to antimicrobial agents is needed to guard against the possible spread of resistant clones.


### Antimicrobial Resistance in *Salmonella* Enteritidis, Southern Italy, 1990-1998

**Antonino Nastasi,* Caterina Mammina,† and Lucia Cannova†**
*University of Florence, Florence, Italy; and
†University of Palermo, Palermo, Italy

During 1990 to 1998, we identified multidrug-resistant isolates of *Salmonella* Enteritidis in southern Italy. Plasmids containing class I integrons and codifying for synthesis of extended-spectrum ß-lactamases were detected. Active surveillance for resistance to antimicrobial agents is needed to guard against the possible spread of resistant clones.

In the last decade, the incidence of *Salmonella* Enteritidis infections has increased in many countries. In Europe, this serotype now predominates among Salmonella isolates from humans (1). In southern Italy, identification of *S.* Enteritidis has increased steadily since 1990, in parallel with increases throughout Europe. After a temporary decline in 1995 and 1996, isolation rates from both sporadic cases and foodborne outbreaks increased. During 1998, records from the Center for Enteric Pathogens in southern Italy show identification rates of approximately 45% in all human Salmonella isolates and 61% in isolates from patients hospitalized for enteritis. In the Enteritidis serotype, resistance to antimicrobial drugs is rare, but resistance to antibacterial agents has been increasing in some Mediterranean countries (2).

We conducted a retrospective study of antimicrobial drug resistance patterns of *S.* Enteritidis isolates identified from human, animal, and environmental sources in southern Italy from 1990 to 1998. We also investigated mechanisms of resistance at the molecular level.

### The Study

From 1990 to 1998, 1,889 strains of *S.* Enteritidis were referred to the Center for Enteric Pathogens, Palermo, southern Italy: 86% were of human origin, 2.9% from infected animals (mainly poultry), 6.7% from sewage plant effluents and surface water, and 4.4% from foods (mainly eggs and egg-based dishes). All strains were biochemically identified by standard tests and were serotyped for somatic and flagellar antigen identification. Phage types were determined with 10 typing phages (3).

Forty-four (2.2%) of the 1,889 strains tested were resistant to at least one antibiotic; we examined patterns of antibiotic resistance, phage types, and plasmid profiles of these 44 strains (Table). Resistance to ampicillin, alone or associated with other ß-lactams, and tetracycline, alone orassociated with aminoglycosides, sulfonamides, and trimethoprim, were the most commonly encountered phenotypes among the *S.* Enteritidis isolates studied. Of the 17 tetracycline-resistant strains, nine and eight, respectively, had transferrable plasmids of 80 and 30 MDal.

Six strains isolated from pediatric patients with enteritis (three in 1992, 1994, and 1996 in Sicily and three in 1997 in Calabria) were resistant to ampicillin, aztreonam, cephalotin, third-generation cephalosporins, and sulfonamides by the Kirby-Bauer method (7). Two of the 1997 isolates were also resistant to chloramphenicol. The double-disk synergy test was positive for all six isolates, suggesting the production of ESBL. In five cases, plasmids of 38, 70, and 80 MDa were shown by conjugation to mediate the complete pattern of resistance. In one strain identified in 1992, a 30-MDal plasmid was detected, but the resistance traits could not be transferred to recipient cells.

Six isolates of *S.* Enteritidis carried integrons with inserted regions of DNA of 0.8 to 2.5 kb (Table). Transconjugant *Escherichia coli* from these strains was also positive, indicating that

Address for correspondence: Caterina Mammina, Via del Vespro 133, I-90127 Palermo, Italy; fax: 39-091-655-3641; e-mail: diptigmi@mbox.unipa.it.

Table. Resistance patterns of *Salmonella* Enteriditis strains, southern Italy, 1990–1998

| Year | Source | Region | Phage types | Resistance pattern[a] | Plasmid pattern (mol. wt., MDa) | Resistance pattern of recipient *Escherichia coli* | Integrons (size of inserted regions, kb) |
|---|---|---|---|---|---|---|---|
| 1990 | human | Sicily | RDNC | Ap | 36, 25 | | |
| 1991 | cake[b] | Sicily | 4 | Su, Tp, Tc | **80**,[c] 36 | Tp, Tc | 2.5 |
| 1992 | seafood | Apulia | 4 | Ap | **30** | Ap | |
| 1992 | seafood | Apulia | 4 | Ap | 36, **30** | Ap | |
| 1992 | human | Sicily | 4 | Ap, Kf, Atm, Caz, Cro, Ctx, Su | 30 | | |
| 1992 | dog | Sicily | RDNC | Ap, Kf, Sm, Su, Tc | **30** | Ap, Sm, Su, Tc | |
| 1992 | human | Calabria | 4 | Gm, Sm, Su | 80, 70 | | 0.8 |
| 1992 | human | Sicily | RDNC | Sm, Su, Tp | **80**, 36 | Sm, Su, Tp | |
| 1992 | human | Calabria | 1 | Su, Tp, Tc | **80**, 36 | Tp, Tc | 1.5 |
| 1993 | human | Calabria | 7 | Gm, Sm, Tc | **80** | Tc | |
| 1993 | human | Sicily | 4 | Sm, Tc | **80**, 36 | Tc | |
| 1993 | human | Sicily | 4 | Sm, Su, Tp, Tc | **80**, 36 | Sm, Su, Tp, Tc | |
| 1993 | human | Sicily | 7 | Sm, Su, Tp, Tc | **80**, 36 | Sm, Su, Tp, Tc | |
| 1994 | human | Sicily | 4 | Ap, Kf, Atm, Caz, Cro, Ctx, Su | **80**, 36 | Ap, Kf, Atm, Caz, Cro, Ctx | 2.0 |
| 1994 | human | Sicily | RDNC | Tc | 36, **30** | Tc | |
| 1994 | human | Sicily | 4 | Tc | 36, **30** | Tc | |
| 1995 | human | Calabria | 4 | Tc | 36, 30 | Tc | |
| 1995 | human | Apulia | 4 | Tc | **80**, 36 | Tc | |
| 1995 | human | Apulia | 7 | Tc | 36, **30** | Tc | |
| 1996 | human | Sicily | 4 | Ap, Kf, Atm, Caz, Cro, Ctx, Su | **80**, 36 | Ap, Kf, Atm, Caz, Cro, Ctx | 2.0 |
| 1996 | human[b] | Sicily | RDNC | Tc | 36, **30** | Tc | |
| 1996 | human | Apulia | RDNC | Tc | **30** | Tc | |
| 1997 | human | Sicily | 4 | Ap | 36, 30 | | |
| 1997 | human | Sicily | 4 | Ap | 36 | | |
| 1997 | human | Sicily | 1 | Ap | 36, 30 | | |
| 1997 | human | Calabria | 4 | Ap, Kf, Atm, Caz, Cro, Ctx, Cm, Su | **70**, 36 | Ap, Kf, Atm, Caz, Cro, Ctx, Cm | |
| 1997 | human | Calabria | 4 | Ap, Kf, Atm, Caz, Cro, Ctx, Cm, Su | **38**, 36 | Ap, Kf, Atm, Caz, Cro, Ctx, Cm | |
| 1997 | human | Calabria | RDNC | Ap, Kf, Atm, Caz, Cro, Ctx, Cm, Su | **80**, 36 | Ap, Kf, Atm, Caz, Cro, Ctx | 2.0 |
| 1997 | human | Apulia | 1 | Ap, Sm, Tc | 36, **30** | Ap, Sm, Tc | |
| 1997 | human | Calabria | 1 | Ap, Sm, Tc | 36, **32** | Ap, Sm, Tc | |
| 1997 | human | Sicily | 4 | Cm, Su, Tp | 36, **32** | Cm, Su, Tp | |
| 1997 | human | Sicily | 4 | Su, Tp | 36 | | |
| 1997 | poultry | Sicily | 14b | Tc | **80**, 36 | Tc | |
| 1997 | human | Sicily | 14b | Tc | **80** | Tc | |
| 1997 | human | Sicily | NT | Tc | **80** | Tc | |
| 1997 | human | Sicily | 13 | Tc | **80**, 36 | Tc | |
| 1997 | human | Sicily | RDNC | Tc, Nal | **80**, 36 | Tc | |
| 1998 | human | Sicily | 4 | Ap | **70**, 36 | Ap | |
| 1998 | sewage | Sicily | RDNC | Ap, Kf | 36 | | |
| 1998 | human | Sicily | RDNC | Tc | 36, **30** | Tc | |
| 1998 | human | Sicily | 7 | Tc | **30** | Tc | |
| 1998 | human | Sicily | 6a | Tc | **80**, 36 | Tc | |
| 1998 | human | Sicily | RDNC | Tc | 36, **30** | Tc | |
| 1998 | poultry | Sicily | RDNC | Tc | **30** | Tc | |

Ap, ampicillin; Kf, cephalotin; Atm, aztreonam; Caz, ceftazidime; Cro, ceftriaxone; Ctx, cefotaxime; Cm, chloramphenicol; Gm, gentamicin; Sm, streptomycin; Su, sulfonamides; Tp, trimethoprim; Tc, tetracycline; Nal, nalidixic acid; RDNC, reaction did not conform; NT, not typable.

[a]The strains were screened for resistance to ampicillin (10 µg), cephalotin (30 µg), cefotaxime (30 µg), chloramphenicol (30 µg), ciprofloxacin (5 µg), gentamicin (10 µg), nalidixic acid (30 µg), streptomycin (10 µg), sulfonamides (300 µg), tetracycline (30 µg), and trimethoprim (5 µg). Strains resistant to cefotaxime were subsequently tested for susceptibility to aztreonam (30 µg), ceftazidime (30 µg), and ceftriaxone (30 µg). Resistance was determined by disk diffusion (4). The double-disk synergy test was performed (4) on strains presumed to produce extended-spectrum ß-lactamase (ESBL). Plasmid DNA was extracted by an alkaline lysis method (5). Electrophoresis on 0.7% agarose gels was performed on samples of plasmid DNA. The approximate molecular weight of plasmids was estimated by comparison with plasmids of known molecular size extracted from *Escherichia coli*. Conjugation experiments were carried out in Luria-Bertani broth. Transconjugant colonies of *E. coli* were selected after growth on MacConkey agar containing rifampin (300 µg/ml) and ampicillin (50 µg/ml), streptomycin (30 µg/ml), chloramphenicol (30 µg/ml), or tetracycline (30 µg/ml). All resistant isolates were screened for class I integrons by a strict protocol with oligonucleotide primers specific for the sequence of the 5'-CS and 3'-CS regions adjacent to the site-specific recombinational insertion sequence (6). Primer sequences were 5'-CS, GGCATCCAAGCAGCAAG and 3'-CS,A AGCAGACTTGACCTGA (5).

[b]Source in outbreak.

[c]Numbers in bold indicate the approximate molecular size of resistance plasmids.

the integrons were carried on plasmids. DNA fragments of approximately 2.0 kb were obtained from ESBL-producing strains.

### Conclusions

During the 9-year study, a small proportion of resistant strains was found within Enteritidis, 2.3% showing resistance to at least one antimicrobial drug and 0.9% to three or more. Prevalence in southern Italy was similar to that in other European countries, such as England and Wales (8) and the Czech Republic (9); however, it was lower than prevalence detected from 1987 to 1993 in Greece, where up to 67.4% of strains of *S.* Enteritidis from human and nonhuman sources were resistant to antibiotics and the resistance rate increased steadily until 1991 (2). No temporal trend or possible association with source was investigated in resistance patterns identified in southern Italy because resistant strains are rare and usually from human sources.

The unusual characteristics of antimicrobial resistance of some *S.* Enteritidis isolates highlight the problem of emergence of drug resistance in a common serotype of Salmonella, transmitted in popular food items and often implicated in foodborne outbreaks. We identified six ESBL-producing isolates from epidemiologically unrelated cases, a rare finding (10-12). All six strains were isolated from community-acquired enteritis cases in otherwise healthy children, who had no recent history of hospitalization or antimicrobial therapy. This observation is not consistent with the hypothesis that multidrug-resistant clones are selected or resistance determinants are acquired as a consequence of antibiotic treatment. Moreover, the presence of integrons in strains isolated as long ago as 1991 is of particular concern because of the ability of these elements to disseminate resistance traits by intra- and inter-specific gene transfer (13,14).

Although most isolates identified in southern Italy were susceptible, some aspects of the epidemiology of *S.* Enteritidis are cause for concern. Active monitoring of *S.* Enteritidis strains for resistance to antibacterial drugs seems crucial because of the public health implications of a potential spread of resistant clones.

Dr. Nastasi is a professor of hygiene at the department of public health of the University of Florence, Italy. He has been director of the Centre for Enteric Pathogens of Southern Italy. His research interests include epidemiology of infectious diseases and molecular epidemiology of infections by enteric pathogens.

### References

1. Fisher J. Salm/Enter-Net records a resurgence in *Salmonella* enteritidis infection through the European Union. Eurosurveillance Weekly 1997;1:June 26.
2. Tassios PT, Markogiannakis A, Vatopoulos AC, Katsanikou E, Velonakis EN, Kourea-Kremastinou J, et al. Molecular epidemiology of antibiotic resistance of *Salmonella* enteritidis during a 7-year period in Greece. J Clin Microbiol 1997;35:1316-21.
3. Ward LR, De Sa JDH, Rowe B. A phage-typing scheme for *Salmonella* enteritidis. Epidemiol Infect 1987;99:291-4.
4. National Committee for Clinical Laboratory Standards. Performance standards for antimicrobial disk susceptibility tests for bacteria that grow aerobically. Approved standard M7-A4. Villanova (PA): The Committee; 1997.
5. Birnboim HC, Doly J. A rapid alkaline extraction procedure for screening recombinant plasmid DNA. Nucleic Acids Res 1979;7:1513-23.
6. Lévesque C, Piché L, Larose C, Roy PH. PCR mapping of integrons reveals several novel combinations of resistance genes. Antimicrob Agents Chemother 1995;39:185-1.
7. Bauer AW, Kirby MMW, Sherris JC, Turck M. Antibiotic susceptibility testing by a standardized single disk method. Am J Clin Pathol 1966;45:493-6.
8. Threlfall EJ, Ward LR, Skinner JA, Rowe B. Increase in multiple antibiotic resistance in nontyphoidal Salmonellas from human in England and Wales: a comparison of data for 1994 and 1996. Microb Drug Resist 1997;3:263-6.
9. Sramova H, Karpiskova R, Dedicova D, Sisak F, Rychlik I. Properties of Salmonella isolates in the Czech Republic. Epidem Mikrobiol Immunol 1999;48:111-6.
10. Cherian BP, Singh N, Charles W, Prabhakar P. Extended-spectrum beta-lactamase-producing *Salmonella* enteritidis in Trinidad and Tobago. Emerg Infect Dis 1999;5:181-2.
11. Blahova J, Lesicka-Hupkova M, Kralikova K, Krcmerry V, Krcmeryova T, Kubonova K. Further occurrence of extended-spectrum-beta-lactamase-producing *Salmonella* enteritidis. J Chemother 1998;10:291-4.
12. Gaillot O, Clement C, Simonet M, Philippon A. Novel transferable beta-lactam resistance with cephalosporinase characteristics in *Salmonella* enteritidis. J Antimicrob Chemother1997;39:85-7.
13. Stokes H, Hall RA. A novel family of potential mobile DNA elements encoding site-specific gene integration functions: integrons. Mol Microbiol 1989;3:1669-83.
14. Rankin SC, Coyne MJ. Multiple antibiotic resistance in *Salmonella* enterica serotype enteritidis. Lancet 1998;351:1740.
